# The Association Between Pulse Wave Velocity and Cerebrospinal Fluid Biomarkers for Alzheimer’s Disease

**DOI:** 10.1002/alz.095634

**Published:** 2025-01-09

**Authors:** Hyena Kim, Ambar Kulshreshtha, Alvaro Alonso, Felicia Goldstein, Matthew Gold, James J. Lah

**Affiliations:** ^1^ Emory University, Atlanta, GA USA; ^2^ Emory University School of Medicine, Atlanta, GA USA

## Abstract

**Background:**

Prior studies have demonstrated a negative correlation between arterial stiffness and cognitive function, suggesting that pulse wave velocity (PWV) could be a promising noninvasive vascular biomarker for detecting cognitive decline. However, the link between PWV and specific Alzheimer’s Disease (AD) cerebrospinal fluid (CSF) biomarkers has not been examined. This cross‐sectional study examines the association between PWV and AD CSF tau/Aβ ratios.

**Method:**

We analyzed the cross‐sectional association between PWV and AD CSF biomarkers in the Emory Healthy Brain study (EHBS). Participants with normal cognition were enrolled in the EHBS, a preclinical AD biomarker discovery project, to identify early signs of cognitive decline. Participants are cognitively normal without functional limitations and are without neurological diagnoses suggesting prodromal or current degenerative disease. Participants had detailed cognitive and vascular tests and consented to lumbar puncture. CSF pTau/Aβ42 and tTau/Aβ42 ratios were converted into binary variables using established cutoff values of 0.022 and 0.26, respectively. We used an established cut‐off for PWV of 6.7 m/s, which indicates higher arterial stiffness. Unadjusted and adjusted analyses for age, race, sex, education, and CVD risk factors were conducted using logistic regression.

**Result:**

Of 751 participants, 69% were female, 84% were White, and 12% were Black. The mean age was 62, and the mean educational attainment was 17. There were 12% of participants with AD CSF biomarker positivity for tTau/Aβ42 and 16% for pTau/Aβ42. In unadjusted models, higher PWV had 103% (OR = 2.03, 95% CI 1.31‐3.14, p‐value, 0.002) greater odds of AD biomarker positivity for tTau/Aβ42 and 76% (OR = 1.76, 95% CI, 1.18‐2.63, p‐value, 0.006) greater odds of AD biomarker positivity for pTau/Aβ42 compared to participants with lower PWV. In the adjusted model, participants with higher PWV had 73% (OR = 1.73, 95% CI 1.09‐2.74, p‐value, 0.02) greater odds of AD biomarker positivity for tTau/Aβ42 and 49% (OR = 1.49, 95% Cl, 0.98‐2.29, p‐value, 0.07) for pTau/Aβ42 compared to participants with lower PWV.

**Conclusion:**

Increased arterial stiffness, as measured by PWV, is associated with AD CSF biomarker positivity. Future longitudinal studies should assess whether PWV is effective for screening and early detection of AD pathology.

